# Effects of an Encapsulated Fruit and Vegetable Juice Concentrate on Obesity-Induced Systemic Inflammation: A Randomised Controlled Trial

**DOI:** 10.3390/nu9020116

**Published:** 2017-02-08

**Authors:** Evan J. Williams, Katherine J. Baines, Bronwyn S. Berthon, Lisa G. Wood

**Affiliations:** Priority Research Centre for Healthy Lungs, Hunter Medical Research Institute, University of Newcastle, Callaghan NSW 2308, Australia; evan.j.williams@uon.edu.au (E.J.W.); katherine.baines@newcastle.edu.au (K.J.B.); bronwyn.berthon@newcastle.edu.au (B.S.B.)

**Keywords:** obesity, systemic Inflammation, fruit and vegetable concentrate, blood lipids

## Abstract

Phytochemicals from fruit and vegetables reduce systemic inflammation. This study examined the effects of an encapsulated fruit and vegetable (F&V) juice concentrate on systemic inflammation and other risk factors for chronic disease in overweight and obese adults. A double-blinded, parallel, randomized placebo-controlled trial was conducted in 56 adults aged ≥40 years with a body mass index (BMI) ≥28 kg/m^2^. Before and after eight weeks daily treatment with six capsules of F&V juice concentrate or placebo, peripheral blood gene expression (microarray, quantitative polymerase chain reaction (qPCR)), plasma tumour necrosis factor (TNF)α (enzyme-linked immunosorbent assay (ELISA)), body composition (Dual-energy X-ray absorptiometry (DEXA)) and lipid profiles were assessed. Following consumption of juice concentrate, total cholesterol, low-density lipoprotein (LDL) cholesterol and plasma TNFα decreased and total lean mass increased, while there was no change in the placebo group. In subjects with high systemic inflammation at baseline (serum C-reactive protein (CRP) ≥3.0 mg/mL) who were supplemented with the F&V juice concentrate (*n* = 16), these effects were greater, with decreased total cholesterol, LDL cholesterol and plasma TNFα and increased total lean mass; plasma CRP was unchanged by the F&V juice concentrate following both analyses. The expression of several genes involved in lipogenesis, the nuclear factor-κB (NF-κB) and 5′ adenosine monophosphate-activated protein kinase (AMPK) signalling pathways was altered, including phosphomevalonate kinase (PMVK), zinc finger AN1-type containing 5 (ZFAND5) and calcium binding protein 39 (CAB39), respectively. Therefore, F&V juice concentrate improves the metabolic profile, by reducing systemic inflammation and blood lipid profiles and, thus, may be useful in reducing the risk of obesity-induced chronic disease.

## 1. Introduction

The global epidemic of obesity, “globesity”, is rapidly becoming a major public health problem in many parts of the world [1]. In the U.S., 78.6 million adults (35% of the population) are obese [2]. Australia is not far behind, with 28% of adults being obese [3]. Obesity develops due to high caloric intake and/or inadequate energy expenditure over time, with genetic susceptibility also likely to have contributed to obesity rates [4]. Overweight and obesity are associated with an epidemic of many chronic diseases, including type 2 diabetes mellitus (T2DM), cardiovascular disease (CVD), stroke, hypertension and certain cancers [1]. 

Obesity is characterised by chronic, low-grade systemic inflammation, due to the release of pro-inflammatory mediators from adipose tissue [5,6]. Adipose tissue from lean individuals contains small, insulin-sensitive adipocytes, as well as tissue-resident macrophages of the alternatively-activated phenotype (M2) [6,7]. With the development of obesity, adipose tissue becomes characterised by large insulin-resistant adipocytes accompanied by the presence of classically-activated macrophages (M1), which are pro-inflammatory [6,7]. Adipocyte hypertrophy and death, adipose tissue hypoxia and changes in immune cell populations alter adipokine secretory patterns [8]. This process leads to the development of insulin resistance [6].

In obese individuals, adipose tissue produces and secretes adipokines, such as leptin and adiponectin, and pro-inflammatory mediators, including tumour necrosis factor (TNF)α [9]. The production and release of TNFα is the first step in a cascade of pro-inflammatory mediator release [10,11]. TNFα is a mediator in energy metabolism, immune function and apoptosis [12] and increases the activation of neutrophils [13]. C-reactive protein (CRP) is another inflammatory mediator that is produced by both adipocytes and the liver, in response to cytokines, such as TNFα and interleukin (IL)-6 [14]. CRP is also positively associated with the degree of obesity [10,11] and is an independent predictor of myocardial infarction risk, stroke and T2DM [10].

Toll-like receptors (TLRs) and nuclear factor-κB (NF-κB)-associated mechanisms have been proposed as primary molecular pathways mediating adipose tissue inflammation [15]. TLRs activate NFκB [15], a transcription factor and potent inducer of gene transcription of pro-inflammatory cytokines. A plethora of inflammatory pathways is activated in obesity with mechanisms, such as, phosphorylation of mitogen-activated protein kinases (MAPKs) [16] and AMP-activated protein kinase (AMPK) activity modulating adipose tissue inflammation [17]. Peroxisome proliferator-activated receptor-α (PPARα), a nuclear receptor primarily expressed in the liver and potent inducer of fat oxidizing genes, has also been reported to reduce adipose tissue inflammation [8,18].

Systemic inflammation puts obese individuals at increased risk of chronic diseases that have an inflammatory pathology, including CVD, diabetes and cancer [19,20]. Weight loss, achieved via energy restriction, is one approach, which has been shown to correct or improve the overall metabolic profile. Weight loss significantly reduces circulating CRP [21,22], TNFα [21,22,23,24,25] and leptin [22]. Indeed, for every 1 kg of weight lost, there is a corresponding reduction in CRP of 0.13 mg/L [26], accompanied by improvements in systemic insulin resistance [27,28]. However, it is well accepted that achieving and maintaining weight loss is unattainable for some individuals. Hence, alternative approaches for improving the metabolic profile of obese individuals are needed.

Many studies, in both humans and animals, demonstrate that the consumption of dietary bioactive compounds found in fruit and vegetables, such as polyphenols and carotenoids, is able to improve the metabolic profile and reduce the risk of chronic diseases via signalling pathways involving NF-κB, MAPKs and AMPK [8]. A recent study in overweight individuals found that a two-fold increase in fruit and vegetable intake for 16 weeks significantly decreased body mass index (BMI) and supine systolic blood pressure and increased plasma concentrations of the antioxidants α-carotene, β-carotene and lutein [29]. Another study found that supplementing healthy individuals with 500 g of strawberries, rich in vitamin C and anthocyanins, daily for one month improved the lipid profile by decreasing total cholesterol, low-density lipoprotein (LDL) cholesterol and triglycerides, as well as decreasing markers of oxidative stress, including plasma malondialdehyde, urinary 8-OHdG and isoprostane levels [30]. There is also emerging evidence that supplements containing dietary bioactive compounds can provide similar benefits. A recent review, including 18 human trials with a total of 1363 adults, reported that fruit and vegetable concentrate supplements significantly increased serum levels of antioxidants (β-carotene, vitamins C and E), as well as folate. In addition, the supplements reduced homocysteine and markers of oxidative stress [31]. 

In this study, we hypothesised that obesity-associated inflammation could be suppressed using a fruit and vegetable concentrate supplement. The aim of this study was to examine the effects of an encapsulated fruit and vegetable juice concentrate on systemic inflammation and other risk factors for chronic disease in overweight and obese adults.

## 2. Materials and Methods 

### 2.1. Study Design 

This was a double-blinded, parallel, randomised, placebo-controlled trial in 56 subjects aged ≥40 years and with a BMI ≥28 kg/m^2^, recruited from March 2014 to September 2014. Subjects were randomised to receive a fruit and vegetable (F&V) concentrate supplement (Juice Plus+^®^ Orchard, Garden and Berry Blends) (*n* = 28) or placebo (*n* = 28) for 8 weeks. Subjects commenced a low fruit and vegetable diet (≤3 serves per day of fruit and vegetables combined) two weeks before randomisation as a washout period, and continued this diet for the duration of the study. Subjects were assessed at commencement of the study (Week 0) and again after supplementation (Week 8) at the Clinical Trials Facility at the Hunter Medical Research Institute. At both visits, anthropometric measures, blood pressure, pulse wave velocity and body composition were measured, and blood samples were collected. Subjects were non-smokers and had an absence of or irregular menses if they were female. Subjects were excluded if they were unwilling or unable to limit the intake of fruit and vegetables to no more than 3 servings/day, if they had used dietary or nutritional supplements within the previous 4 weeks, if they were current smokers, were currently participating in a weight management program or actively attempting to lose weight, were currently using any medication known to significantly influence inflammation, had an allergy to the supplement ingredients or chronic excessive alcohol consumption, which is associated with a high risk of chronic health problems, defined as ≥43 standard drinks per week for men and ≥29 drinks per week for women [32]. Unused capsules were collected at the end of the study to determine adherence with the intervention. Participants with adherence of >85% were included in the analysis, determined by the pill count back method.

### 2.2. Randomization

Subjects were randomly allocated to the treatment or placebo group. Subjects were screened to determine eligibility, and those eligible were assigned a unique study number according to the randomisation schedule. The randomisation schedule was computer generated with blocks of variable size and stratified by BMI and gender. Randomisation was managed by an independent statistician at the Hunter Medical Research Institute. During the treatment phase, both the subjects and the investigators were blinded to the allocation.

### 2.3. Study Supplement

The F&V concentrate capsules contained a blended fruit, vegetable and berry juice powder concentrate derived from the following: acerola, cherry, apple, bilberry, blackberry, black currant, blueberry, beetroot, broccoli, cabbage, carrot, concord grape, cranberry, elderberry, kale, orange, peach, papaya, parsley, pineapple, raspberry, red currant, spinach and tomato (Juice Plus+^®^ Orchard, Garden and Berry Blends) as described previously [33]. Briefly, the F&V concentrate capsules provided; β-carotene 3.05 mg/day, α-tocopherol 4.8 mg/day, vitamin C 300 mg/day, folate 350 mg/day and polyphenols ~600 mg/day [34]. The placebo capsules were identical in appearance, opaque white capsules containing microcrystalline cellulose. All subjects were instructed to take three capsules twice daily with meals, in agreement with the label use instructions for the retail product, for a total of six capsules per day.

### 2.4. Ethics

The study was conducted according to the guidelines laid down in the Declaration of Helsinki, and all procedures involving human subjects were approved by the Hunter New England Health Human Research Ethics Committee (14/02/19/3.01) and registered with the University of Newcastle Human Research Ethics Committee. Written informed consent was obtained from all subjects. The trial was prospectively registered with the Australian New Zealand Clinical Trials Registry (ANZCTRN12614000079640).

### 2.5. Anthropometric Measures and Quality of Life

Anthropometric assessments (height and weight) were performed following an overnight fast. Body mass index (BMI) was calculated as body weight (kg)/(height (m))^2^. Height (metres) was measured using the stretch stature method to 2 decimal places using a wall-mounted stadiometer (Seca 220, Seca, Hamburg, Germany). Body weight (kg) was measured to 1 decimal place with subjects wearing light clothing and without shoes, using calibrated electronic scales. Quality of life was assessed by the short form health survey (SF-36) questionnaire [35]. Waist circumference (WC) was measured to the nearest 0.1 cm at the midpoint between the lower costal edge and the iliac crest, using a non-extensible steel tape (Lufkin W606PM, Apex Tool Group, Sparks, MD, USA).

### 2.6. Blood Pressure

A blood pressure cuff was placed firmly around the upper arm of the participant, centred over the brachial artery. After resting quietly in a seated position for 10 min, four consecutive blood pressure and heart rate readings were taken at one-minute intervals by a single observer using an electronic vital signs monitor (6000 Series, Welch Allyn, Skaneateles Falls, NY, USA). The first reading was discarded and an average of the remaining measurements recorded for analysis.

### 2.7. Dual Energy X-ray Absorptiometry

Body composition was measured using a dual energy X-ray absorptiometry (DXA) machine and associated software (DXA Lunar Prodigy; Encore 2007 Version 11.40.004, GE Medical Systems, Madison, WI, USA). Total and regional absolute and percentage of fat and lean mass were calculated (kg). 

### 2.8. Peripheral Blood Biomarkers

Fasting blood was collected into ethylenediaminetetraacetic acid (EDTA) tubes, then centrifuged at 3000× *g*, at 10 °C for 10 min. Plasma was separated and stored at −80 °C for batched analysis at the end of the study. TNFα, soluble (s)TNF receptor (R) 1 and sTNFR2 were measured by Human Quantikine ELISA Kits (R&D Systems, Minneapolis, Minnesota, USA). Oxidised (ox)-LDL was also analysed by ELISA (Mercodia, Uppsala, Sweden). Serum CRP, total cholesterol, LDL cholesterol, high-density lipoprotein (HDL) cholesterol, triglycerides and haemoglobin A1c (HbA1c) were measured in peripheral blood by an accredited pathology service (Hunter Area Pathology Service, Newcastle, NSW, Australia).

### 2.9. Plasma Antioxidant Levels

Plasma carotenoids (α-carotene, β-carotene, lutein, lycopene, β-cryptoxanthin) and plasma α-tocopherol were analysed by reverse phase high-performance liquid chromatography (HPLC) (Agilent LC 1200 System, Agilent Technologies, Santa Clara, CA, USA) using methods established in our laboratory [36,37,38]. Briefly, ethanol:ethyl acetate (1:1) containing internal standards (canthaxanthin and α-tocopherol acetate) and butylated hydroxytoluene were added to the sample. The solution was centrifuged (3000× *g*, 48 °C, 5 min), and the supernatant was collected; this was repeated 3 times adding ethyl acetate twice and then hexane to the pellet. Ultrapure water was then added to the pooled supernatant fluid, and the mixture was centrifuged. The supernatant was then decanted, and the solvents were evaporated with nitrogen. The sample was then reconstituted in dichloromethane:methanol (1:2). Chromatography was performed on a Hypersil ODS column with a flow rate of 0.3 mL/min, using the mobile phase of acetonitrile:dichloromethane:methanol 0.05% ammonium acetate (85:10:5). Carotenoids and tocopherols were detected at 450 nm and 290, respectively, using a photodiode array.

### 2.10. Dietary Analysis

Usual dietary intake over the previous 12 months was assessed at Week 0 by food frequency questionnaire using the Dietary Questionnaire for Epidemiological Studies v2 (DQES) (Victorian Cancer Council [39]). A 24-h food recall was also recorded at Week 0 and Week 8 by a dietitian and analysed using nutrient analysis software (FoodWorks Version 7, Xyris Software P/L, Kenmore Hills, QLD, Australia). Serving sizes were those as defined by the National Health and Medical Research Council Australian Dietary Guidelines [40]. Briefly, 75 grams of vegetables are equivalent to one serving and 150 grams of fruit counted as one serving.

### 2.11. Microarray Analysis

Microarray analysis was conducted in the F&V concentrate group only, to detect differences in gene expression before and after the intervention. Whole blood RNA was extracted from PAXgene tubes using the PAXgene Blood RNA Extraction Kit (Qiagen, Hilden, Germany), as per the manufacturer’s instructions. RNA was quantitated and the quality assessed using Bioanalyser and Quant-iT Ribogreen RNA Reagent (Molecular Probes, Invitrogen, Carlsbad, CA, USA); the average RNA integrity number was 8.07. Whole genome gene expression was determined in 500 ng of blood RNA, which was amplified using the Illumina TotalPrep RNA Amplification Kit (Ambion, Waltham, MA, USA). Seven hundred fifty nanograms of amplified RNA were then hybridised to HumanHT-12 Version 4 Expression BeadChip (Illumina, San Diego, CA, USA). Microarrays were then scanned using the Illumina Bead Station. The microarray primary data used in this study are available at the national centre for biotechnology information (NCBI) Gene Expression Omnibus [41] under Accession Number GSE87454. 

### 2.12. PCR Analysis

Real-time PCR testing was performed on 2 key genes, which demonstrated the greatest fold difference in expression and had biological relevance to the study hypothesis. Two hundred nanograms of extracted RNA (see above) were converted to cDNA using the High Capacity cDNA Reverse Transcription Hit (Applied Biosystems, Foster city, CA, USA). Standard Taqman methods were used. Taqman qPCR primer and probes of target genes were purchased in kit form (Applied Biosystems, Foster city, CA, USA). Target gene expression was measured relative to the housekeeping gene 18S, and the stability value of 18S was calculated as 0.045 using NormFinder [42]. All reactions were carried out using the ABI 7500 Real-Time PCR Machine (Applied Biosystems, Foster city, CA, USA). Taqman Assay IDs are as follows; phosphomevalonate kinase (PMVK) (Hs00559915_m1) and zinc finger AN1-type containing 5 (ZFAND5) (Hs04400278_g1).

### 2.13. Sample Size and Statistical Analysis

Sample size was determined using the Power and Sample Size calculation programme (PS Version 3.0.43, Vanderbilt University, Nashville, TN, USA) [43]. Based on our previous studies of the expression of multiple genes involved in inflammatory pathways, we required *n* = 26 subjects per group to have 80% power to detect a mean difference in expression of genes of 20%, with standard deviation (SD) = 25%. Our initial recruitment target of *n* = 64 allowed for 20% dropouts; however, recruitment was ceased at *n* = 61, as a very low dropout rate of 8% was achieved.

Per protocol analysis was conducted in 2 stages; Part 1: analysis of the full cohort; Part 2: analysis of the subset of individuals who are at increased risk of chronic disease due to high systemic inflammation levels at baseline, defined as serum CRP ≥3.0 mg/mL. The normality of all data was assessed using the D’Agostino–Pearson omnibus normality test. 

Baseline data: demographics, biomarkers and dietary intake were compared using the unpaired Student’s *t*-test (parametric data) or Mann–Whitney U-test (non-parametric data). 

Intervention results: following the intervention, within-group differences were compared using paired *t*-tests for parametric data or the Wilcoxon rank sum test for non-parametric data. Between-group differences were analysed using analysis of covariance (ANCOVA) to test for differences between treatment groups after adjusting for baseline values. Associations between variables were assessed using Pearson’s correlation for normally distributed variables and Spearman’s rank correlation coefficient for non-parametric data.

Microarray whole genome gene expression analysis: data were exported to RStudio Version 3.2.2 (RStudio, Boston, MA, USA) using Illumina’s Genome Studio 3.0 (Illumina, San Diego, CA, USA) and processed using the R packages lumi [44] and limma [45]. Data were normalised via log transformation and the baseline converted to the median of all samples. Data were filtered based on whether the gene was detected as present in 28 or more samples, as this was half the samples available from the intervention group. Gene profiles were analysed for differential expression by paired *t*-test for significance and fold change in RStudio Version 3.2.2. 

## 3. Results

### 3.1. Part 1: Analysis of Full Cohort

#### 3.1.1. Participant Flow

**Figure 1:**
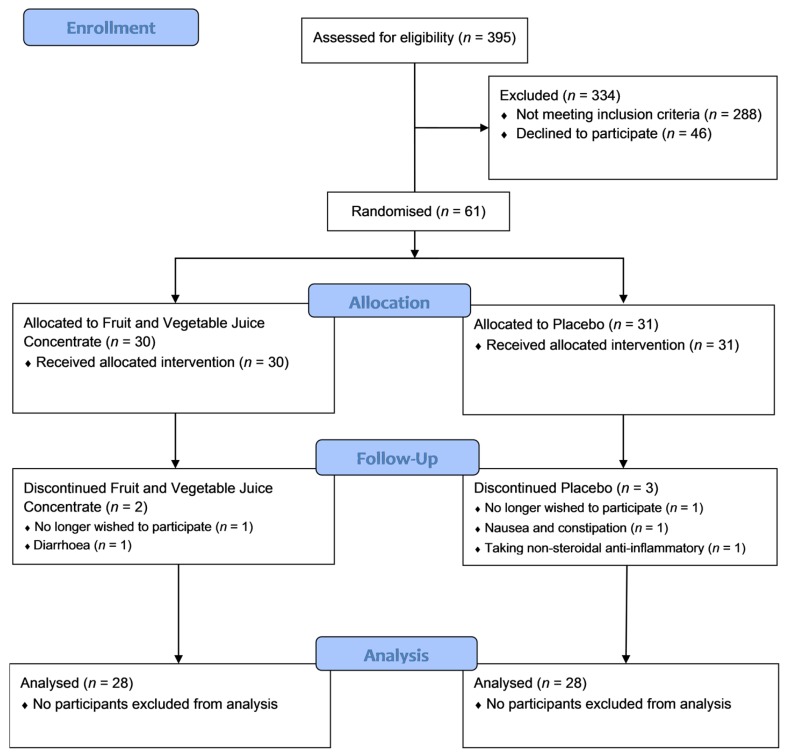
Patient flow consolidated standards of reporting trials (CONSORT) diagram. A total of 395 patients were screened for eligibility to participate; 61 of those who were eligible and wished to participate were randomised to either the fruit and vegetable juice concentrate intervention group or placebo, each taking three capsules twice daily.

Sixty-one subjects were randomised, with five withdrawing after visit 1 (Figure 1). Of those who withdrew, one was suffering from constipation and fluid retention, one was suffering from diarrhoea and stomach pain, one had difficulty swallowing the study medication, one required anti-inflammatory treatment for a prior knee injury and one no longer wished to participate in the study. Of the 56 subjects who completed the study, 28 were in the F&V concentrate group and 28 were in the placebo group. The mean adherence rate was 97.7%.

#### 3.1.2. Subject Demographics, Dietary Intake and Plasma Nutrient Levels

At baseline, there were no differences between groups in demographics or nutrient intakes (Table 1). Daily intake of fruit and vegetables was also not different at baseline between the groups and was well below the recommended levels (Table 2). Fruit and vegetable intake did not change during the intervention (Table 2). After eight weeks, plasma β-carotene and total carotenoids increased significantly within the F&V concentrate group and compared to the placebo group (Table 2). Plasma lycopene levels decreased in the placebo group compared to the F&V concentrate group.

#### 3.1.3. Lipid Profile, Glycated Haemoglobin and Systemic Inflammatory Markers

Total cholesterol and LDL cholesterol decreased within the F&V concentrate group only, while triglycerides increased in the placebo group. Comparison of the two groups showed that only the change (Δ) in triglycerides was different between the F&V concentrate and placebo groups. Glycated haemoglobin (HbA1c) was unchanged after the intervention in both the F&V concentrate and placebo groups (Table 3). Plasma TNFα decreased following the F&V concentrate supplementation, and the difference in ΔTNFα between the F&V concentrate and placebo groups approached significance (*p* = 0.071). There was a negative correlation between Δβ-carotene and ΔTNFα (*r* = −0.352, *p* = 0.018). Plasma sTNFR2 concentration increased in the placebo group, while plasma sTNFR1, ox-LDL and CRP were unchanged in the F&V concentrate and placebo groups at Week 8 (Table 3).

#### 3.1.4. Body Composition, Blood Pressure and Quality of Life before and after the Intervention 

Weight, BMI and waist circumference did not change in both the F&V concentrate and placebo groups (Table 4). Total lean mass increased within the F&V concentrate group, and the change compared to the placebo group approached significance (*p* = 0.057). Systolic blood pressure was significantly decreased after eight weeks in both the F&V concentrate and placebo groups; however, the difference between groups was not significant. Diastolic blood pressure, pulse and quality of life (SF36) were not changed after the F&V concentrate supplementation or the placebo (Table 4).

### 3.2. Part 2: Analysis of a Subgroup with High Baseline CRP (≥3.0 mg/mL)

#### 3.2.1. Subject Demographics, Dietary Intake and Plasma Nutrient Levels

To determine whether the effects of the F&V concentrate supplement were more evident in subjects with elevated systemic inflammation, a subgroup analysis was performed in subjects with baseline CRP ≥3.0 mg/mL. By using this criteria, the number of participants in the F&V concentrate and placebo groups was reduced to *n* = 17 and *n* = 15, respectively. The baseline demographics and dietary intakes of these groups were analysed (Table 5) and found to be similar, except for BMI, which was higher in the placebo group. Baseline daily intake of fruit and vegetables was not different between the groups and was well below recommended levels. Fruit and vegetable intake did not change during the intervention (Table 6). Plasma β-carotene and total carotenoids were both increased within the F&V concentrate group compared to the placebo group at Week 8. Lycopene was significantly decreased within the placebo group compared to the F&V concentrate group (Table 6).

#### 3.2.2. Blood Lipids, Glycated Haemoglobin and Systemic Inflammatory Markers

Cholesterol and LDL cholesterol were significantly decreased within the F&V concentrate group, but were not different compared to the placebo group (Table 7). Triglycerides, HDL cholesterol, total cholesterol/HDL ratio and HbA1c were unchanged within either the F&V concentrate or placebo groups. Plasma levels of TNFα were significantly decreased within the F&V concentrate group and also compared to the placebo group (Table 7). The soluble receptors, sTNFR1 and sTNFR2, were significantly different between groups following the intervention, due to an increase in concentration in the placebo group. Oxidised-LDL and CRP were unchanged in both groups (Table 7).

#### 3.2.3. Body Composition, Blood Pressure and Quality of Life Before and after the Intervention 

At the completion of the trial, weight, BMI, waist circumference, blood pressure and quality of life were unchanged in both groups (Table 8). Total lean mass and pulse rate significantly increased within the F&V concentrate group, but were not different compared to the placebo group.

#### 3.2.4. Peripheral Blood Gene Expression

In the subgroup of participants with high baseline CRP (*n* = 16), microarray analysis revealed that 1632 genes were differentially expressed after supplementation with the F&V concentrate. One thousand one hundred forty six genes were upregulated, while 486 genes were downregulated, with the highest absolute fold change being 1.44 and the lowest fold change being 1.03. 

**Figure 2:**
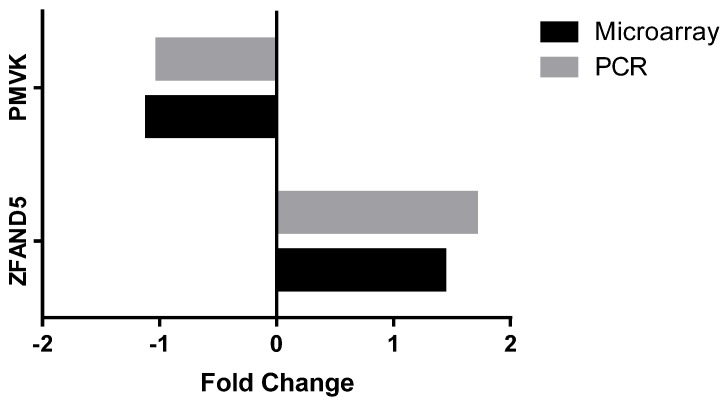
Validation of microarray analysis; fold changes in gene expression following the intervention as determined by microarray versus quantitative polymerase chain reaction (qPCR). Zinc finger, AN1-type domain 5 (ZFAND5); Phosphomevalonate kinase (PMVK).

**Figure 3:**
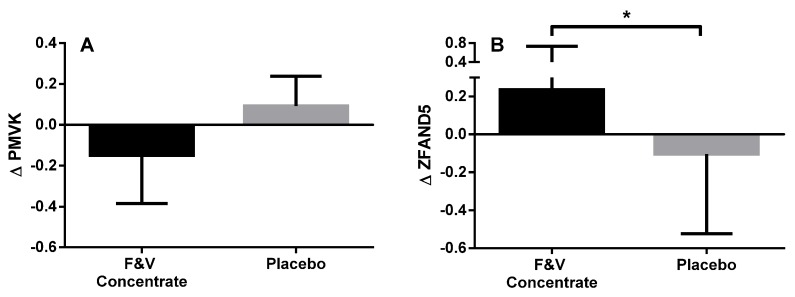
Change in peripheral blood mRNA expression of PMVK (**A**) and ZFAND5 (**B**) at baseline compared to Week 8 in the fruit and vegetable juice concentrate (F&V concentrate) and placebo groups; measured using qPCR; the data presented are the median (IQR); the statistical test used was the Mann–Whitney test; * *p* < 0.05 versus baseline.

From the genes that were significantly differentially expressed, several genes were identified that are involved in biologically-relevant signalling pathways, including: lipogenesis, NF-kB and AMPK pathways (Table 9). Pathways analysis was also performed on genes that were differentially expressed with a *p* < 0.05, using gene ontology tool to help explain relationships (GATHER) [46]. The upregulated genes were found to be involved in five different pathways, those being: the insulin signalling pathway, neuroactive ligand-receptor interaction, adherens junction, regulation of the actin cytoskeleton and the wnt signalling pathway. There were 18 upregulated genes involved in the insulin signalling pathway: calmodulin 1 (CALM1), casitas B-lineage lymphoma (CBL), crk-like protein (CRKL), hexokinase 2 (HK2), insulin receptor substrate 2 (IRS2), mitogen-activated protein kinase kinase 1 (MAP2K1), mitogen-activated protein kinase 6 (MAPK6), phosphodiesterase 3B (PDE3B), phosphorylase kinase regulatory subunit beta (PHKB), phosphatidylinositol-4, 5-biphosphate 3-kinase catalytic subunit beta isoform (PIK3CB), protein phosphatase 1 catalytic subunit gamma (PPP1CC), 5′-AMP-activated protein kinase subunit gamma-2 (PRKAG2), cAMP-dependent protein kinase type I-alpha regulatory subunit (PRKAR1A), tyrosine-protein phosphatase non-receptor type 1 (PTPN1), glycogen phosphorylase, liver form (PYGL), raf-1 proto-oncogene, serine/threonine kinase (RAF1), ras homolog enriched in brain (RHEB), ras-related protein R-Ras2 (RRAS2). Two gene targets for qPCR analysis were chosen to validate the microarray data; ZFAND5 and PMVK. Figure 2 shows that ZFAND5 and PMVK mRNA expression had similar fold changes following the intervention when assessed using both microarray and qPCR. As seen in the microarray analysis, gene expression of ZFAND5 as determined by PCR analysis was also significantly upregulated following supplementation with the F&V concentrate. While PMVK expression was downregulated in the microarray analysis, the downregulated expression in the PCR analysis was not statistically significant (Figure 3).

## 4. Discussion

In this study, we found that in older subjects who are overweight or obese, F&V concentrate supplementation led to decreases in total cholesterol, LDL cholesterol, TNFα and systolic blood pressure. We conducted a secondary analysis of the subgroup of participants who had high systemic inflammation at baseline, as we hypothesised that these participants were more likely to obtain an improvement in metabolic health as a result of the supplement. Similar results were observed in the subgroup analysis, with F&V concentrate supplementation leading to a decrease in total cholesterol, LDL cholesterol and TNFα, and indeed, the size of the effects was greater. A number of genes associated with lipogenesis, AMPK and NF-κB signalling pathways were differentially expressed following the intervention. Pathways analysis also revealed that several genes involved in the insulin signalling pathway were upregulated in the subgroup of participants with high baseline CRP. 

Total and LDL cholesterol were both found to be significantly decreased after the F&V concentrate intervention with greater changes seen in the subgroup with high baseline CRP. A recent Cochrane review used meta-analyses to demonstrate that increased fruit and vegetable consumption can reduce cholesterol levels [47]. Included studies achieved an average increase in fruit and vegetable intake of 1.88 (95% confidence interval (CI): 1.07 to 2.70) servings per day and total blood cholesterol levels reduced by 0.11 mmol/L (95% CI: −0.19 to −0.03). We observed a much greater decrease in total cholesterol in the high baseline CRP subgroup in this study. Importantly, the size of the decreases in total and LDL cholesterol that were observed in this study are substantial and clinically important. In the full cohort analysis, we observed a 0.2 mmol/L (3.5%) reduction in total cholesterol, which is estimated to be equivalent to a weight loss of 4 kg and an 8% to 9% reduction in CVD risk [48,49]. We also observed a 0.13 mmol/L (3.5%) decrease in LDL cholesterol, estimated to be equivalent to a 6.5 kg weight loss and 5% reduction in CVD risk [48,49]. In subjects who had elevated systemic inflammation at baseline, we observed a 0.45 mmol/L (7.4%) reduction in total cholesterol, which is estimated to be equivalent to a weight loss of 9 kg and an 18% to 19% reduction in CVD risk [48,49]. In this group, we also observed a 0.16 mmol/L (4.0%) decrease in LDL cholesterol, estimated to be equivalent to an 8 kg weight loss and a 4% reduction in CVD risk [48,49].

Serum triglycerides were found to be unchanged by the F&V concentrate in both the full cohort analysis and the high baseline CRP subgroup analysis. However, we did observe an inverse correlation between Δβ-carotene and Δtriglycerides. There is previous evidence that F&V concentrate supplementation and polyphenol-rich diets can lower triglyceride levels in overweight boys with elevated baseline triglycerides [50] and in overweight or obese adults [51]. It is likely that F&V concentrate supplementation would have the greatest effect in individuals with elevated triglycerides, which may explain the lack of change in this study, as the majority of subjects had normal triglyceride levels at baseline. Interestingly, in the placebo group, triglycerides significantly increased in the full cohort analysis after the intervention. This may be related to the decrease in total carotenoids that was observed in the placebo group after eight weeks, which indicates that bioactive compounds continued to washout during the intervention period. This may have also masked the effect of the F&V concentrate, if dietary sources of carotenoids and other phytochemicals were continuing to washout during the intervention phase.

Microarray analysis revealed differential expression of several key genes involved in lipogenesis following the F&V concentrate supplementation. *PMVK* (or phosphomevalonate kinase) was found to be significantly downregulated. *PMVK* catalyses conversion of mevalonate 5-phosphate into mevalonate 5-diphosphate, which is the fifth reaction of the cholesterol biosynthetic pathway [52]. Similarly, gene expression of farnesyl diphosphate synthase (*FDPS*) was downregulated. This may also lead to reduced cholesterol production as the gene encodes an enzyme that catalyses the production of farnesyl diphosphate (*FPP*), a key biosynthetic intermediate used in the formation of cholesterol [53]. Interestingly, a recent study has shown that supplementation with the same F&V concentrate used in this study led to increased levels of circulating fatty acid binding protein 4 (FABP4), possibly via modulation of PPAR activity, resulting in altered lipid metabolism and accumulation [54]. Hence, there are several mechanisms by which the F&V concentrate may have led to the reduced blood lipid levels that were observed in this study.

Following intervention with the F&V concentrate, there was a small, but statistically-significant reduction in TNFα in the full cohort analysis, with a much greater reduction seen in the high baseline CRP subgroup analysis. TNFα is a cell signalling protein involved in systemic inflammation, with a primary role of regulating immune cells. Previous studies have shown that increasing fruit and vegetable intake reduces systemic inflammation, specifically CRP concentration [55,56], while a previous study of F&V concentrate supplementation showed that monocyte chemotactic protein 1 (MCP-1), macrophage inflammatory protein 1b (MIP-1b) and regulated on activation, normal T cell expressed and secreted (RANTES) were reduced [57]. The F&V concentrate used in this study is rich in β-carotene, which has been shown to reduce inflammatory mediator production, including TNFα [58,59]. For example, a study in mice found that β-carotene reduced the inflammatory response to LPS, including TNFα production [59]. Another study in a non-alcoholic fatty liver model in rats found that TNFα levels were markedly ameliorated with the administration of β-carotene [58]. Hence, the potential for carotenoids to reduce inflammation has previously been demonstrated.

TNFR1 and TNFR2 are the receptors to TNFα. Upon binding, inflammation is induced via various pathways, including activation of NF-kB and MAPK pathways. In this study, TNFR2 increased in the placebo group, in both the full cohort analysis and the subgroup analysis. Again, we speculate that this may have been a result of the decrease in total carotenoids that occurred in the placebo group during the intervention period, which suggests that the two-week washout period was not long enough to washout background levels of carotenoids prior to the intervention period. 

A number of key genes involved in adenosine monophosphate-activated protein kinase (AMPK) and NF-κB signalling were found to be differentially expressed following intervention with F&V concentrate, and these may have contributed to the anti-inflammatory effect that we observed. AMPK is a key regulator of energy metabolism homeostasis, as AMPK inhibits energy consuming activities, such as protein, fatty acid and cholesterol synthesis, and stimulates energy production through glucose and lipid catabolism [60]. AMPK activation can also inhibit NF-κB signalling, thus reducing inflammation [60], and impaired AMPK activity has been shown to lead to insulin resistance [61,62]. AMPK signalling genes that were upregulated following the F&V concentrate intervention that have known anti-inflammatory effects include: *IRS2*, *CAB39* and *SIRT1*. Previously, a study found that knockdown of *IRS2* expression resulted in mice developing insulin resistance due to increased inflammation [63], while another study in mice found that AMPK signalling activated by *CAB39* inhibits NF-κB activation [64]. SIRT1, a downstream target of AMPK, inhibits NF-κB signalling, thus reducing inflammation [60,65,66,67]. Other genes that were upregulated in our analysis include *BCL2* and *TNF-AIP3*, which are known inhibitors of NF-κB signalling [68,69,70,71,72,73,74,75,76,77]. Another gene found to be upregulated following intervention with the F&V concentrate was ZFAND5, also known as ZNF216 [78]. A previous study on ZFAND5 found that it was an inhibitor of NF-κB activation, by reducing TNFα, IL-1 and TLR4 in a dose-dependent manner [79]. Hence, our data show several pathways by which the F&V concentrate appears to be having an anti-inflammatory effect, with several AMPK and NF-κB signalling pathway genes contributing to this effect. 

F&V concentrate supplementation has previously been shown to reduce homeostatic model assessment-insulin resistance (HOMA-IR) in a group of overweight boys [50]. While there was no change in HbA1C in the intervention group in our study, pathways analysis using GATHER found that a number of genes involved in the insulin signalling pathway were upregulated following intervention with the F&V concentrate. In the high baseline CRP subgroup analysis, the increased expression of the genes *HK2, IRS2, PDE3B, PHKB, PRKAG2, PTPN1* and *RHEB* suggests that the intervention with the F&V concentrate increases insulin sensitivity, as these genes are all involved in reducing blood glucose levels via the insulin signalling pathway [80,81,82,83,84,85,86]. This provides further evidence of the ability of the F&V concentrate to improve the metabolic profile of obese individuals.

Following the intervention with the F&V concentrate, systolic blood pressure (SBP) was found to be reduced in the full cohort analysis. A previous observational study found there was an inverse relationship between β-carotene levels and SBP [87]. In another intervention study, increasing fruit and vegetable consumption to at least five daily portions significantly reduced SBP [88]. This reduction in SBP indicates a decreased risk of cardiovascular diseases, such as myocardial infarction, stroke and congestive heart failure [15]. Interestingly, however, there was also a significant reduction in SBP in the placebo group. Hence, we cannot rule out the possibility that subjects in the trial experienced the ‘white coat effect’ prior to their baseline visit, with their blood pressure becoming elevated due to anxiety about attending the research centre for the first time [89].

F&V concentrate supplementation led to an increase in total lean mass in both the full cohort analysis and the high baseline CRP subgroup analysis, likely due, at least in part, to the decrease in TNFα that we observed. TNFα plays a central role in muscle wasting [90]. Circulating TNFα binds to peripheral muscle cell receptors, stimulating the production of reactive oxygen species (ROS) and cellular apoptosis. In addition, the receptor binding stimulates NF-κB activation, possibly enhanced by ROS. The result is protein loss, caused directly via increased ubiquitin activity and indirectly via decreased myogenic differentiation (MyoD) expression, which decreases myofibril synthesis [90]. This observation suggests that F&V concentrate supplementation may be beneficial in settings where muscle wasting is undesirable, e.g., during aging or cachexia. 

Baseline intake of fruit and vegetables was well below recommended levels for both the F&V concentrate and placebo groups. Following the intervention with the F&V concentrate, β-carotene and total carotenoids were significantly increased in the full cohort analysis, as well as the high baseline CRP subgroup analysis. α-tocopherol was found to be unchanged by the F&V concentrate intervention. The F&V concentrate used in this study provides a daily dose of 3.5 mg β-carotene and 4.8 mg α-tocopherol. These daily doses correspond approximately to 100% of the usual daily intake of β-carotene, but only ~50% of the usual daily intake of α-tocopherol [3]. Hence, it is not surprising that we observed an increase in circulating levels of β-carotene, but not α-tocopherol. The supplement also contains polyphenols (at least 119 compounds), including ellagitannins, gallotannins, dihydrochalcones, flavan-3-ols including proanthocyanidins, flavanones, flavones, flavonols, anthocyanins, hydroxybenzoic acids, hydroxycinnamic acids, phenylethanoids and lignans [34]. Whilst we were unable to analyse the circulating levels of these compounds or their metabolites in the samples from this study, it is likely that they would have contributed to the effects that we observed, as the dose delivered (~600 mg/day) is approaching the usual daily dose for most populations [34]. As previously discussed, total carotenoids decreased in the placebo group following the eight-week intervention. This suggests that the two-week run-in period on the low fruit and vegetable washout diet was not long enough, and hence, carotenoids continued to decrease during the eight-week intervention period. Importantly, if the washout period was not adequate, this would also have affected the results in the intervention group, as the potential benefits of the supplement may have been masked, as background levels of carotenoids continued to wash out throughout the intervention period. In future trials, a longer wash out period is recommended. 

## 5. Summary and Conclusions

To summarise, this randomised controlled trial in obese, older individuals shows that F&V concentrate supplementation has the potential to improve the metabolic profile of overweight and obese individuals by reducing blood lipid levels and systemic inflammation, as well as improving body composition. The size of the improvements is clinically significant, as the reduction in total cholesterol that we observed in the full cohort is estimated to be equivalent to a weight loss of 4 kg and an 8% to 9% reduction in CVD risk [48,49]. In the subset of participants who had elevated systemic inflammation at baseline, the reduction in total cholesterol was equivalent to a 9 kg weight loss and an 18% to 19% reduction in CVD risk [48,49]. Interestingly, while significant changes were seen in the whole group of participants, the changes were more pronounced in subjects who had elevated CRP levels at baseline. Hence, while all obese individuals are likely to gain some benefit from supplementation with F&V concentrate, those with high baseline systemic inflammation or blood lipids appear likely to obtain the greatest improvements. We conclude that in obese individuals, who typically have a low fruit and vegetable intake, F&V concentrate supplementation may be beneficial for improving the metabolic profile, thus reducing the risk of developing chronic inflammatory disease.

## Figures and Tables

**Table 1:** Baseline subject demographics and nutrient intake. F&V, fruit and vegetables.

	F&V Concentrate (*n* = 28)	Placebo (*n* = 28)	*p*-Value
**Demographics**			
Gender (male/female)	11/17	13/15	0.788
Age (years) ^a^	61.4 ± 1.5	57.9 ± 1.4	0.091
BMI (kg/m^2^) ^a^	34.6 ± 0.7	37.0 ± 1.3	0.271
Smoking Status (never/ex)	14/14	16/12	0.592
**Nutrient Intake**			
Total Energy (KJ/day) ^b^	7785 (6145, 10,371)	6986 (5573, 8448)	0.110
Total fat (g/day) ^b^	78 (63, 107)	70 (59, 87)	0.229
SFA (g/day) ^b^	32 (25, 45)	28 (24, 37)	0.205
PUFA (g/day) ^a^	12 ± 1	12 ± 1	0.626
MUFA (g/day) ^a^	31 ± 2	28 ± 2	0.286
Protein (g/day) ^a^	101 ± 7	77 (73, 99)	0.103
Carbohydrates (g/day) ^a^	202 ± 15	177 ± 11	0.169
Fibre (g/day) ^a^	23 ± 2	21 ± 1	0.285
Calcium (mg/day) ^a^	965 ± 50	857 ± 50	0.134
Folate (µg/day) ^a^	342 ± 17	238 ± 14	0.046
Iron (mg/day) ^b^	14 (10, 21)	11 (9, 14)	0.103
Magnesium (mg/day) ^a^	319 ± 21	270 ± 14	0.060
Niacin (mg/day) ^b^	22 (17, 29)	18 (14, 25)	0.126
Phosphorus (mg/day) ^a^	1732 ± 118	1459 ± 77	0.058
Potassium (mg/day) ^b^	3078 (2312, 3534)	2409 (2065, 3014)	0.051
Retinol (µg/day) ^b^	351 (303, 443)	340 (277, 435)	0.651
Riboflavin (mg/day) ^a^	3 ± 0	2 ± 0	0.055
Sodium (mg/day) ^a^	2647 ± 197	2476 ± 158	0.500
Thiamine (mg/day) ^a^	2 ± 0	1 ± 0	0.064
Vitamin C (mg/day) ^a^	105 ± 11	95 ± 8	0.417
Vitamin E (mg/day) ^a^	7 ± 1	6 ± 0	0.273
Zinc (mg/day) ^b^	13 (9, 18)	10 (9, 12)	0.114
α-carotene (µg/day) ^b^	811 (416, 1244)	643 (324, 830)	0.122
β-carotene (µg/day) ^a^	4068 ± 387	3255 ± 273	0.092
β-cryptoxanthin (µg/day) ^b^	125 (48, 284)	169 (85, 234)	0.675
Lutein/zeaxanthin (µg/day) ^a^	862 ± 78	723 ± 65	0.177
Lycopene (µg/day) ^b^	3967 (2602, 6338)	3807 (2324, 7564)	0.950

BMI: body mass index; SFA: saturated fatty acids; MUFA: monounsaturated fatty acids; PUFA: polyunsaturated fatty acids; ^a^ mean ± standard error of the mean (SEM); ^b^ median (Q1, Q3).

**Table 2:** Fruit and vegetable intake, plasma carotenoids and plasma α-tocopherol before and after the intervention.

	F&V Concentrate (*n* = 28)			Placebo (*n* = 28)			
	0	8 Weeks	*p*-Value *	0	8 Weeks	*p*-Value *	ANCOVA ^ǂ^ *p*-Value
**Fruit and Vegetable Intake (Servings/Day)**							
Fruit ^b^	1.00 (0.00, 1.88)	0.75 (0.00, 1.00)	0.517	1.00 (0.00, 1.00)	0.50 (0.00, 1.00)	0.149	0.222
Vegetables ^a^	1.45 ± 0.19	1.52 ± 0.18	0.784	1.34 ± 0.20	1.46 ±. 023	0.683	0.945
Total ^a^	2.38 ± 0.21	2.30 ± 0.16	0.788	2.14 ± 0.23	2.00 ± 0.23	0.665	0.383
**Plasma Carotenoids (mg/L)**							
Lutein ^b^	0.43 (0.36, 0.52)	0.45 (0.34, 0.59)	0.545	0.37 (0.30, 0.52)	0.37 (0.28, 0.49)	0.922	0.127
β-cryptoxanthin ^b^	0.09 (0.05, 0.15)	0.07 (0.05, 0.12)	0.375	0.07 (0.04, 0.16)	0.06 (0.03, 0.13)	0.200	0.269
Lycopene ^b^	0.10 (0.08, 0.14)	0.12 (0.08, 0.18)	0.213	0.11 (0.07, 0.17)	0.08 (0.04, 0.11)	0.007	0.005
α-carotene ^b^	0.01 (0.00, 0.15)	0.01 (0.00, 0.01)	0.188	0.01 (0.00, 0.02)	0.01 (0.00, 0.01)	0.018	0.095
β-carotene ^b^	0.11 (0.07, 0.15)	0.16 (0.11, 0.26)	<0.001	0.07 (0.00, 0.16)	0.06 (0.00, 0.11)	0.127	<0.001
Total Carotenoids ^b^	0.73 (0.60, 1.05)	0.85 (0.76, 0.99)	0.017	0.72 (0.51, 1.01)	0.59 (0.49, 0.80)	0.019	<0.001
**Plasma α-Tocopherol (mg/L) ^b^**	13.6 (11.3, 15.9)	14.2 (12.1, 15.4)	0.849	14.3 (11.3, 25.6)	14.4 (11.6, 17.7)	0.360	0.568

* The *p*-value refers to the change between baseline and eight weeks within each group; ^ǂ^ analysis of covariance (ANCOVA) was used to compare the change in the F&V concentrate group to the change in the placebo group; ^a^ mean ± standard error of the mean (SEM); ^b^ median (Q1, Q3).

**Table 3:** Lipid profiles, glycated haemoglobin and systemic inflammation before and after the intervention.

	F&V Concentrate (*n* = 28)			Placebo (*n* = 28)			
	0	8 Weeks	*p*-Value *	0	8 Weeks	*p*-Value *	ANCOVA ^ǂ^ *p*-Value
**Total Cholesterol (mmol/L)**	5.70 (5.00, 6.30)	5.50 (4.83, 6.15)	0.015	5.90 (4.50, 6.70)	5.60 (4.60, 6.20)	0.532	0.359
**LDL Cholesterol (mmol/L)**	3.63 (3.17, 4.36)	3.50 (2.95, 4.27)	0.032	4.15 (3.03, 4.63)	3.51 (2.89, 4.10)	0.089	0.904
**HDL Cholesterol (mmol/L)**	1.20 (1.10, 1.40)	1.20 (1.10, 1.30)	0.815	1.20 (1.00, 1.40)	1.20 (1.00, 1.40)	0.941	0.308
**Total/HDL Cholesterol**	4.50 (4.00, 5.10)	4.55 (4.05, 5.00)	0.221	4.60 (3.90, 5.60)	4.60 (3.90, 5.50)	0.587	0.634
**Triglycerides (mmol/L)**	1.24 (0.86, 1.81)	1.23 (1.01, 1.67)	0.344	1.36 (0.97, 1.83)	1.53 (1.06, 1.84)	0.012	0.022
**HbA1c (%)**	5.40 (5.20, 5.60)	5.30 (5.10, 5.50)	0.570	5.40 (5.20, 5.70)	5.30 (5.10, 5.50)	0.149	0.407
**TNFα (pg/mL)**	1.04 (0.87, 1.41)	1.02 (0.55, 1.41)	0.037	1.07 (0.79, 1.22)	0.94 (0.82, 1.26)	0.797	0.071
**sTNFR1 (pg/mL)**	1140 (1017, 1382)	1108 (992, 1335)	0.829	1120 (967, 1319)	1147 (953, 1383)	0.378	0.668
**sTNFR2 (pg/mL)**	2479 (2257, 3034)	2345 (2209, 3130)	0.467	2332 (1986, 2677)	2547 (2047, 2754)	0.006	0.299
**Ox-LDL (mU/L)**	48,620 (41,249, 62,976)	48,352 (41,937, 57,960)	0.990	50,053 (41,262, 64,661)	48,911 (43,400, 59,733)	0.551	0.781
**CRP (mg/mL)**	3.1 (1.7, 5.1)	3.9 (1.2, 5.9)	0.536	3.2 (1.7, 5.2)	2.5 (1.5, 5.4)	0.769	0.301

* The *p*-value refers to the change between baseline and eight weeks within each group; ^ǂ^ ANCOVA was used to compare the change in the F&V concentrate group to the change in the placebo group; all data are presented as the median (Q1, Q3). Low-density lipoprotein (LDL); high-density lipoprotein (HDL); haemoglobin A1c (HbA1c); tumour necrosis factor (TNF) α; soluble TNF receptor 1 (sTNFR1); soluble TNF receptor 2 (sTNFR2); oxidized-LDL (ox-LDL); C-reactive protein (CRP).

**Table 4:** Body composition, blood pressure and quality of life before and after the intervention.

	F&V Concentrate (*n* = 28)			Placebo (*n* =28)			
	0	8 Weeks	*p*-Value *	0	8 Weeks	*p*-Value *	ANCOVA ^ǂ^ *p*-Value
**BMI (kg/m^2^) ^a^**	34.6 ± 0.7	34.6 ± 0.8	0.076	37.0 ± 1.3	36.1 ± 1.2	0.781	0.336
**Waist circumference (cm) ^a^**	113.7 ± 2.2	112.5 ± 2.3	0.649	116.6 ± 2.8	116.0 ± 2.9	0.863	0.699
**Body Composition**							
Total Body Fat (kg) ^a^	42.6 ± 1.7	42.7 ± 1.7	0.728	45.1 ± 2.4	44.8 ± 2.5	0.231	0.618
% Body Fat ^a^	46.7 ± 1.5	46.4 ± 1.5	0.168	45.0 ± 1.5	44.4 ± 1.6	0.384	0.134
Total Lean Mass (kg) ^a^	49.3 ± 2.4	50.0 ± 2.4	0.018	54.4 ± 2.0	55.0 ± 2.1	0.836	0.057
% Lean Mass ^a^	51.4 ± 1.4	51.9 ± 1.4	0.078	53.5 ± 1.5	54.0 ± 1.5	0.560	0.096
Android: Gynoid Fat Ratio ^a^	1.2 ± 0.0	1.2 ± 0.0	0.499	1.1 ± 0.0	1.1 ± 0.0	0.270	0.649
**Systolic BP (mmHg) ^a^**	131.7 ± 2.3	125.9 ± 1.9	0.005	136.7 ± 2.8	132.4 ± 2.3	0.037	0.110
**Diastolic BP (mmHg) ^a^**	80.6 ± 1.2	79.0 ± 1.0	0.097	82.6 ± 1.0	82.3 ± 1.0	0.665	0.055
**Pulse (BPM) ^a^**	66.1 ± 1.7	68.6 ± 1.7	0.175	67.8 ± 1.7	66.4 ± 1.6	0.709	0.178
**Quality of Life (SF-36)**							
Physical ^a^	47.2 ± 1.6	47.8 ± 1.5	0.788	46.6 ± 1.9	49.2 ± 1.6	0.678	0.682
Mental ^b^	51.6 (43.6, 58.7)	54.1 (48.3, 59.1)	0.211	56.4 (40.1, 59.8)	54.0 (45.3, 58.1)	0.897	0.290

BMI: body mass index; BP: blood pressure; * the *p*-value refers to the change between baseline and eight weeks within each group; ^ǂ^ ANCOVA was used to compare the change in the F&V concentrate group to the change in the placebo group; ^a^ mean ± SEM; ^b^ median (Q1, Q3). 36 item short form health survey (SF-36).

**Table 5:** Baseline subject demographics and nutrient intake of subjects with high baseline CRP (≥3.0 mg/mL).

	F&V Concentrate (*n* = 16)	Placebo (*n* = 15)	*p*-Value
**Demographics**			
Gender (male/female)	3/13	6/9	0.252
BMI (kg/m^2^) ^a^	35.6 ± 1.1	40.2 ± 1.5	0.016
Age (years) ^a^	60.8 ± 1.5	56.6 ± 2.0	0.097
Smoking Status (never/ex)	9/8	8/7	1.000
**Nutrient Intake**			
Total Energy (KJ/day) ^a^	7884 ± 792	7569 ± 625	0.758
Total fat (g/day) ^a^	83 ± 9	81 ± 7	0.857
SFA (g/day) ^b^	30 (24, 45)	28 (24, 43)	0.688
PUFA (g/day) ^a^	11 ± 1	12 ± 1	0.609
MUFA (g/day) ^a^	30 ± 3	30 ± 3	>0.999
Protein (g/day) ^b^	94 (67, 120)	77 (73, 110)	0.858
Carbohydrates (g/day) ^a^	190 ± 20	181 ± 16	0.738
Fibre (g/day) ^a^	22 ± 2	20 ± 2	0.429
Calcium (mg/day) ^a^	926 ± 64	871 ± 67	0.559
Folate (**µ**g/day) ^b^	267 (194, 329)	213 (189, 294)	0.418
Iron (mg/day) ^b^	12 (9, 20)	10 (9, 14)	0.509
Magnesium (mg/day) ^a^	312 ± 31	271 ± 20	0.271
Niacin (mg/day) ^b^	20 (14, 25)	18 (14, 26)	0.800
Phosphorus (mg/day) ^b^	1500 (1250, 2002)	1357 (1140, 1859)	0.377
Potassium (mg/day) ^b^	2847 (2090, 3609)	2501 (2081, 2896)	0.533
Retinol (**µ**g/day) ^b^	342 (254, 507)	376 (311, 473)	0.688
Riboflavin (mg/day) ^b^	2 (2, 3)	2 (2, 3)	0.397
Sodium (mg/day) ^b^	2177 (1797, 2738)	2286 (1845, 3623)	0.463
Thiamine (mg/day) ^b^	2 (1, 2)	1 (1, 2)	0.558
Vitamin C (mg/day) ^b^	88 (63, 111)	90 (57, 111)	0.883
Vitamin E (mg/day) ^a^	7 ± 1	6 ± 1	0.702
Zinc (mg/day) ^b^	13 (8, 16)	10 (9, 14)	0.716
α-Carotene (**µ**g/day) ^a^	916 ± 128	684 ± 121	0.198
β-Carotene (**µ**g/day) ^a^	351 ± 36	3350 ± 412	0.352
β-Cryptoxanthin (**µ**g/day) ^b^	105 (48, 193)	164 (89, 225)	0.222
Lutein/zeaxanthin (**µ**g/day) ^b^	803 (584, 903)	744 (332, 1134)	0.509
Lycopene (**µ**g/day) ^b^	3523 (2409, 4931)	2829 (2040, 9169)	0.887

BMI: body mass index; SFA: saturated fatty acids; MUFA: monounsaturated fatty acids; PUFA: polyunsaturated fatty acids; ^a^ mean ± SEM; ^b^ median (Q1, Q3).

**Table 6:** Fruit and vegetable intake, plasma carotenoids and plasma α-tocopherol before and after the intervention in subjects with high baseline CRP (≥3.0 mg/mL).

	F&V Concentrate (*n* = 16)			Placebo (*n* = 15)			
	0	8 Weeks	*p*-Value *	0	8 Weeks	*p*-Value *	ANCOVA ^ǂ^ *p*-Value
**Fruit and Vegetable Intake (Servings/Day)**							
Fruit ^b^	1.00 (0.00, 1.00)	0.75 (0.00, 2.00)	0.899	0.75 (0.00, 1.38)	0.50 (0.00, 1.00)	0.475	0.283
Vegetables ^b^	1.25 (0.63, 2.00)	1.75 (1.00, 2.00)	0.606	1.00 (0.13, 1.88)	1.00 (0.50, 2.00)	0.435	0.893
Total ^a^	2.13 ± 0.26	2.38 ± 0.23	0.478	1.78 ± 0.25	2.00 ± 0.32	0.596	0.475
**Plasma Carotenoids (mg/L)**							
Lutein ^b^	0.41 (0.37, 0.47)	0.45 (0.33, 0.55)	0.519	0.36 (0.29, 0.42)	0.35 (0.28, 0.40)	0.855	0.204
β-cryptoxanthin ^b^	0.08 (0.05, 0.13)	0.07 (0.05, 0.12)	0.900	0.07 (0.02, 0.13)	0.06 (0.02, 0.13)	0.500	0.491
Lycopene ^b^	0.10 (0.08, 0.15)	0.12 (0.08, 0.14)	0.850	0.11 (0.09, 0.19)	0.10 (0.03, 0.12)	0.003	0.026
α-carotene ^b^	0.01 (0.00, 0.02)	0.01 (0.00, 0.01)	0.148	0.01 (0.00, 0.02)	0.00 (0.00, 0.02)	0.059	0.410
β-carotene ^b^	0.11 (0.06, 0.14)	0.15 (0.10, 0.26)	0.003	0.09 (0.00, 0.17)	0.00 (0.00, 0.15)	0.393	0.002
Total Carotenoids ^b^	0.72 (0.60, 0.88)	0.89 (0.61, 0.98)	0.035	0.72 (0.44, 0.94)	0.54 (0.48, 0.80)	0.066	<0.0001
**Plasma α-Tocopherol (mg/L) ^b^**	14.1 (12.5, 16.3)	14.8 (13.6, 16.7)	0.677	14.5 (12.8, 17.2)	14.5 (11.8, 17.3)	0.761	0.826

* The *p*-value refers to the change between baseline and eight weeks within each group; ^ǂ^ ANCOVA was used to compare the change in the F&V concentrate group to the change in the placebo group; ^a^ mean ± SEM; ^b^ median (Q1, Q3).

**Table 7:** Blood lipids, glycated haemoglobin and systemic inflammation before and after the intervention in subjects with high baseline CRP (≥3.0 mg/mL).

	F&V Concentrate (*n* = 16)			Placebo (*n* = 15)			
	0	8 Weeks	*p*-Value *	0	8 Weeks	*p*-Value *	ANCOVA ^ǂ^ *p*-Value
**Total Cholesterol (mmol/L)**	6.10 (5.30, 6.45)	5.65 (5.40, 6.20)	0.016	6.30 (4.70, 6.70)	5.95 (4.95, 6.43)	0.538	0.549
**LDL Cholesterol (mmol/L)**	3.98 (3.37, 4.48)	3.82 (3.34, 4.33)	0.016	4.21 (2.87, 4.67)	3.91 (3.22, 4.50)	0.279	0.854
**HDL Cholesterol (mmol/L)**	1.30 (1.10, 1.40)	1.20 (1.13, 1.38)	0.656	1.20 (1.00, 1.40)	1.20 (1.00, 1.40)	0.941	0.804
**Total/HDL Cholesterol**	4.90 (3.95, 5.40)	4.80 (4.40, 5.08)	0.324	5.20 (4.30, 5.90)	4.80 (3.95, 5.73)	0.398	0.944
**Triglycerides (mmol/L)**	1.30 (0.98, 2.24)	1.41 (1.17, 1.82)	0.520	1.58 (1.16, 1.85)	1.77 (1.09, 1.91)	0.268	0.205
**HbA1c (mmol/mol)**	36.0 (32.5, 38.0)	34.0 (33.0, 37.0)	0.197	36.5 (33.8, 40.3)	36.0 (33.0, 37.5)	0.783	0.662
**HbA1c (%)**	5.40 (5.15, 5.60)	5.30 (5.13, 5.48)	0.128	5.45 (5.28, 5.83)	5.40 (5.20, 5.55)	0.629	0.284
**TNFα (pg/mL)**	1.13 (0.95, 1.52)	0.95 (0.70, 1.40)	0.007	1.07 (0.79, 1.30)	0.96 (0.87, 1.26)	0.632	0.035
**sTNFR1 (pg/mL)**	1140 (980, 1491)	1143 (988, 1414)	0.324	1294 (1047, 1371)	1347 (1176, 1442)	0.212	0.031
**sTNFR2 (pg/mL)**	2698 (2371, 3152)	2389 (2205, 3005)	0.198	2554 (2313, 2881)	2711 (2532, 2998)	0.002	0.009
**Ox-LDL (mU/L)**	48,833 (43,191, 63,536)	50,719 (42,229, 61,437)	0.357	52,683 (39,688, 64,661)	45,559 (43,009, 62,543)	0.536	0.852
**CRP (mg/mL)**	4.8 (3.6, 9.4)	5.2 (3.8, 6.6)	0.930	5.0 (4.1, 7.4)	5.4 (3.8, 7.1)	0.820	0.861

* The *p*-value refers to the change between baseline and eight weeks within each group; ^ǂ^ ANCOVA was used to compare the change in the F&V concentrate group to the change in the placebo group; all data are presented as the median (Q1, Q3).

**Table 8:** Body composition, blood pressure and quality of life before and after the intervention in subjects with high baseline CRP (≥3.0 mg/mL).

	F&V Concentrate (*n* = 16)			Placebo (*n* = 15)			
	0	8 Weeks	*p*-Value *	0	8 Weeks	*p*-Value *	ANCOVA ^ǂ^ *p*-Value
**BMI (kg/m^2^) ^a^**	35.6 ± 1.1	35.5 ± 1.2	0.263	40.2 ± 1.5	40.3 ± 1.5	0.721	0.531
**Waist circumference (cm) ^a^**	113.8 ± 3.4	113.5 ± 3.7	0.480	123.4 ± 3.9	124.3 ± 3.6	0.806	0.939
**Body Composition**							
Total Body Fat (kg) ^a^	45.5 ± 2.3	44.6 ± 2.4	0.362	52.9 ± 2.7	53.2 ± 2.8	0.345	0.421
% Body Fat ^a^	49.9 ± 1.7	48.9 ± 1.8	0.062	47.7 ± 1.7	47.8 ± 1.7	0.615	0.069
Total Lean Mass (kg) ^a^	46.1 ± 3.0	46.9 ± 3.2	0.049	58.1 ± 3.1	58.2 ± 3.2	0.919	0.221
% Lean Mass ^a^	48.5 ± 1.6	49.5 ± 1.7	0.077	50.7 ± 1.6	50.7 ± 1.6	0.811	0.115
Android: Gynoid Fat Ratio ^a^	1.1 ± 0.0	1.1 ± 0.0	0.892	1.1 ± 0.0	1.1 ± 0.0	0.951	0.996
**Systolic BP (mmHg) ^a^**	131.8 ± 3.5	129.1 ± 2.8	0.243	140.1 ± 3.3	134.0 ± 3.4	0.052	0.953
**Diastolic BP (mmHg) ^a^**	80.3 ± 1.8	80.0 ± 1.1	0.999	84.7 ± 1.6	83.5 ± 1.2	0.371	0.306
**Pulse (BPM) ^b^**	67.0 (61.5, 71.8)	71.0 (67.0, 78.0)	0.021	68.0 (63.0, 71.0)	69.0 (62.0, 72.0)	0.988	0.138
**Quality of Life (SF-36)**							
Physical ^a^	45.9 ± 2.4	46.4 ± 2.0	0.862	44.4 ± 2.4	44.4 ± 2.1	0.843	0.665
Mental ^a^	48.8 ± 3.3	53.7 ± 2.3	0.076	48.6 ± 3.4	49.6 ± 2.9	0.596	0.259

BMI: body mass index; BP: blood pressure; * the *p*-value refers to the change between baseline and eight weeks within each group; ^ǂ^ ANCOVA was used to compare the change in the F&V concentrate group to the change in the placebo group; ^a^ mean ± SEM; ^b^ median (Q1, Q3).

**Table 9:** Microarray analysis: biologically-relevant genes that were differentially regulated following F&V concentrate supplementation in subjects with high baseline CRP (≥3.0 mg/mL).

Gene	Gene Name	Gene Function	Fold Change	*p*-Value
**Lipogenesis**				
**PMVK**	Phosphomevalonate kinase	Catalyses the conversion of mevalonate 5-phosphate into mevalonate 5-diphosphate, the fifth reaction of the cholesterol biosynthetic pathway	−1.102	0.005
**FDFT1**	Farnesyl-diphosphate farnesyltransferase 1	First specific enzyme in cholesterol biosynthesis, catalyses dimerization farnesyl diphosphate to form squalene	1.08	0.014
**FDPS**	Farnesyl diphosphate synthase	Catalyses the production of intermediates in cholesterol biosynthesis	−1.06	0.034
**NF-κB**				
**ZFAND5**	Zinc finger, AN1-type domain 5	Inhibits TNF, IL-1 and TLR-induced NF-κB activation	1.438	0.005
**ATM**	ATM serine/threonine kinase	Regulates tumour suppressor and DNA repair genes	1.242	0.006
**PTGS2**	Prostaglandin-endoperoxide synthase 2	Key enzyme in prostaglandin biosynthesis	1.199	0.042
**PARP1**	Poly (adenosine diphosphate (ADP)-ribose) polymerase 1	Differentiation, proliferation and tumour transformation	1.194	0.008
**PLCG2**	Phospholipase C, gamma 2 (phosphatidylinositol-specific)	Catalyses the conversion of 1-phosphatidyl-1D-myo-inositol 4,5-bisphosphate to 1D-myo-inositol 1,4,5-trisphosphate (IP3) and diacylglycerol (DAG), important second messenger molecules	1.161	0.019
**BCL2**	B-cell chronic lymphoid leukemia (CLL)/lymphoma 2	Important anti-apoptotic protein, classified as an oncogene	1.141	0.003
**TLR4**	Toll-like receptor 4	Pattern recognition receptor implicated in LPS signal transduction	1.118	0.017
**MAP3K7**	Mitogen-activated protein kinase kinase kinase 7	Forms complex with Transforming growth factor beta activated kinase (TAB)-1 or TAB2 which is required for NF-κB	1.112	0.042
**PRKCQ**	Protein kinase C, theta	Important for T-cell activation and NF-κB transcription factor activation	1.082	0.020
**PRKCB**	Protein kinase C, beta	Phosphorylates protein targets involved in B cell activation, apoptosis, endothelial cell proliferation, intestinal sugar absorption	1.072	0.048
**MALT1**	Mucosa-associated lymphoid tissue lymphoma translocation protein 1	Proteolytic activity, many targets involved in regulation of inflammation	1.067	0.028
**TNFAIP3**	Tumour necrosis factor-induced protein 3	Induced by TNF, inhibits NF-κB and TNF-mediated apoptosis	1.059	0.042
**AMPK**				
**CAB39**	Calcium binding protein 39	Stimulates STK11 activity, which is an upstream kinase of AMPK	1.418	0.004
**RAB10**	RAB10; RAS oncogene family	Regulates intracellular vesicle trafficking	1.279	0.020
**SIRT1**	Sirtuin 1	Involved in regulating AMPK expression	1.152	0.019
**IRS2**	Insulin receptor substrate 2	Mediates effects of insulin, insulin-like growth factor 1, and cytokines	1.137	0.009
**RHEB**	Ras homolog enriched in brain	involved in the mechanistic targeting of rapamycin (mTOR) pathway and the regulation of the cell cycle	1.119	0.048
**MAP3K7**	Mitogen-activated protein kinase kinase kinase 7	Forms complex with TAB1 or TAB2, which is required for NF-κB	1.112	0.042
